# Trends in HPV and HPV Vaccine Awareness among Gay and Bisexual Males in the U.S

**DOI:** 10.3390/vaccines10040604

**Published:** 2022-04-13

**Authors:** Ikechukwu Chidobem, Fan Tian, Esther Ogbuokiri, Francis Mgbodile, Chisom Mgbodile, Tahereh Orouji Jokar, Muhammad Ahsan Shah, Frantz Pierre-Louis

**Affiliations:** 1 Department of Medicine, St. Mary’s General Hospital, Passaic, NJ 07055, USA; drchykes@gmail.com (F.M.); chisommgbodile@gmail.com (C.M.); oroujit@gmail.com (T.O.J.); 2 Department of Mathematics, Tufts University, Medford, MA 02155, USA; fan.tian@tufts.edu; 3 Rutgers New Jersey Medical School, Newark, NJ 07103, USA; eo281@njms.rutgers.edu; 4 Faculty of Agriculture and Environment, The Islamia University of Bahawalpur, Bahawalpur 63100, Pakistan; ahsanshahg52@gmail.com; 5 Division of Infectious Diseases, St. Mary’s General Hospital, Passaic, NJ 07055, USA; frantzjr@gmail.com

**Keywords:** HPV, HPV awareness, HPV vaccine, HPV vaccine awareness, gay, bisexual

## Abstract

Human papillomavirus (HPV) is the most common sexually transmitted infection in the United States (US). It is often discussed within the context of women’s sexual health due to its association with cervical cancer. However, HPV is also associated with other cancers and conditions which affect men. Gay and bisexual males (GBM) in the US have higher risks of HPV infection and a higher incidence of HPV-associated anal cancer than heterosexual males. In addition, GBM in the US have a higher prevalence of some high-risk strains of HPV than in other regions. HPV vaccination is highly effective at preventing HPV-associated cancers and genital warts. Several resources have been directed towards improving HPV awareness in the US over the past couple of years to improve vaccination rates. Given the low rates of HPV vaccination among GBM in the US, this study aimed to assess the trends in HPV and HPV vaccine awareness using a nationally representative sample of GBM. We found an overall increase in HPV and HPV vaccine awareness between 2017 and 2020. However, the sociodemographic differences in awareness levels highlight the need for more interventions to improve vaccination rates, especially in this high-risk population.

## 1. Introduction

Human papillomavirus (HPV) is the most common sexually transmitted infection in the United States (US) [1]. While there is no cure for HPV infections, vaccination is highly effective at preventing HPV-related diseases. HPV awareness and vaccination are encouraged by health experts and covered in many health-based campaigns. Due to its long-established association with cervical cancer, HPV is often discussed within the context of women’s sexual health. However, HPV is also a cause for concern in men. The prevalence of oral HPV and high-risk oral HPV infections has been higher in men than women, except for Asian adults [2]. Additionally, genital HPV and high-risk genital HPV infections are higher in men than women [2]. Of the oropharyngeal cancers linked to HPV, male patients represent roughly 80% of those affected [3]. Although the United States (US) Advisory Committee on Immunization Practices (ACIP) initially recommended HPV vaccination for only adolescent females, this recommendation was expanded to include males in 2011 [1]. The currently available 9-valent HPV vaccine (against HPV types 6, 11, 16, 18, 31, 33, 45, 52, and 58) is approved by the US Food and Drug Administration (FDA) for males and females aged 9–45 years [4].

Gay and bisexual males (GBM) are at increased risk for sexually transmitted infections, including HPV. Gay, bisexual, and other men who have sex with men (MSM) are 17 times more likely to get anal cancer than heterosexual men [5]. It has been suggested that more than 50% of HIV-negative GBM have an anogenital HPV infection [6]. The incidence of anal cancer in HIV-negative GBM has been estimated at 35 cases per 100,000 population, in contrast to 1.6 cases per 100,000 population among all men in the U.S. [6,7]. Other studies have also found that anal HPV prevalence among MSM is at least twice as high as among men who exclusively have sex with women [8,9,10]. Another recent study showed that men who have sex with men and transgender women in the US had a higher prevalence of HPV 16 (56%) and HPV 6 (69%) compared to other regions [11]. HPV16 is the most common HPV type detected in HPV-positive noncervical cancers, while HPV6 has been unequivocally linked to HPV-associated genital warts and recurrent respiratory papillomatosis [12].

HPV vaccination rates among the GBM sub-populations have been low among those eligible for vaccination in the United States [5,13]. Another US-based study found that only 13% of GBM aged 18–26 had received any doses of the HPV vaccine [6]. HPV-related awareness has been shown to improve vaccination, and a myriad of legislative measures and resources have been directed towards increasing awareness in the US [14,15,16]. The President’s Cancer Panel labeled HPV vaccine under-utilization as an area of public health concern in 2014 and made recommendations to increase vaccine uptake in the US [14,17]. The recommendations included employing efforts towards the utilization of communication strategies to improve awareness of the benefits, efficacy, and safety of the HPV vaccine [14,17]. Despite these efforts, a recent national study showed declining awareness of HPV and the HPV vaccine within the US general population [14]. 

The authors of this study are not aware of any other studies assessing the trends in HPV and HPV vaccine awareness in GBM in the US over time. This study aims to help fill this literature gap using a nationally representative sample. The findings could help inform efforts toward reducing HPV infections and associated cancers in this high-risk population [6].

## 2. Materials and Methods

### 2.1. Data Source and Study Population

The study used data from the National Cancer Institute’s (NCI) Health Information National Trends Survey (HINTS). HINTS is a nationally representative survey of noninstitutionalized individuals within the US general population aged at least 18 years [18,19]. Data used in this study were obtained from HINTS 5, cycle 1; HINTS 5, cycle 2; HINTS 5, cycle 3; and HINTS 5, cycle 4. Data for these cycles were collected in 2017 (HINTS 5, cycle 1); 2018 (HINTS 5, cycle 2); 2019 (HINTS 5, cycle 3), and 2020 (HINTS 5, cycle 4). All cycles utilized a two-stage sampling strategy [20].

Although the first HINTS cycle was in 2003 (HINTS 1), the HINTS questionnaires did not include questions about sexual orientation until a special round of HINTS data collection in 2015, which was a collaboration between the NCI and the Food and Drug Administration (FDA), called HINTS-FDA. However, that cycle did not assess awareness about HPV or the HPV vaccine. The first HINTS cycle to include questions regarding sexual orientation, HPV awareness, and HPV vaccine awareness in the same survey was in 2017. When carrying out this study, the most recent HINTS survey data was from 2020. To achieve the aims of our study, we reported results from the subgroup of male respondents who self-identified as gay or bisexual, as determined by their responses to the sexual orientation question, “Do you think of yourself as…”. This comprised 61 GBM in 2017 (total number of respondents was 3285); 60 GBM in 2018 (total number of respondents was 3504); 100 GBM in 2019 (total number of respondents was 5438); and 67 GBM in 2020 (total number of respondents was 3865).

### 2.2. Outcome Variables

During the study period (2017–2020), awareness of HPV and the HPV vaccine were assessed, respectively, using the questions “Have you ever heard of HPV? HPV stands for Human Papillomavirus. It is not HIV, HSV, or herpes”, and “A vaccine to prevent HPV infection is available and is called the cervical cancer vaccine or HPV shot. Before today, have you ever heard of the cervical cancer vaccine or HPV shot?”. Possible responses to both questions were “yes” or “no” [20]. 

### 2.3. Independent Variables

The independent variables we used were age, race/ethnicity, annual household income, and education. Age was grouped as 18–34, 35–49, 50–64, and 65 or older. Race/ethnicity was classified as non-Hispanic white, non-Hispanic black, Hispanic, and others. Household annual income was classified as less than $35,000, $35,000–$49,999, $50,000–$74,999, and $75,000 or higher. Education level was categorized as high-school graduate or less, post-high school or some college, college graduate or postgraduate.

### 2.4. Statistical Analysis

All statistical analyses were conducted using R programming (version 4.1.2). The awareness of HPV and the HPV vaccine were calculated as percentages with associated 95% confidence intervals (CI). The calculations for all valid data for all years were stratified by sociodemographic characteristics. Sample weights were performed to ensure the validity of responding sample inferences to the general population. Replicate weights were calculated using the delete one jackknife (JK1) replication method [20]. To account for the replication weights, the R package “srvyr” was used [21]. 

## 3. Results

**Figure 1:**
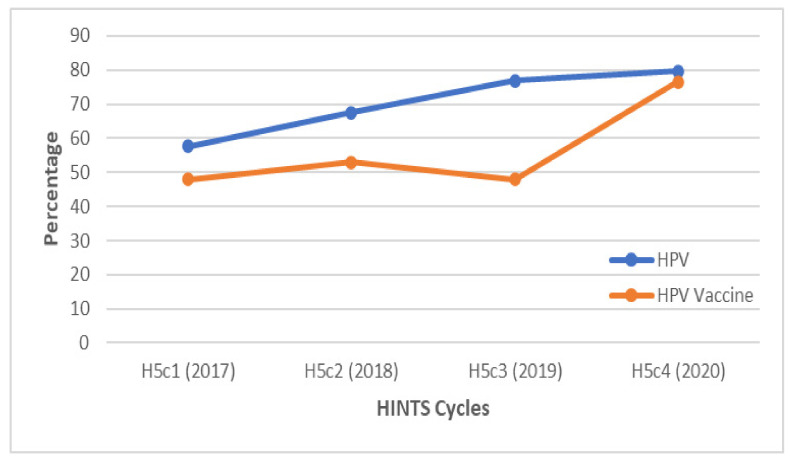
Overall awareness of HPV and the HPV vaccine among gay and bisexual males between 2017 and 2020. The awareness of HPV was consistently greater than the awareness of the HPV vaccine. Overall, the awareness of both was higher in 2020 compared to 2017.

Overall, awareness of HPV among GBM has steadily increased between 2017 and 2020. Awareness of the HPV vaccine increased overall in 2018 compared to 2017. However, it decreased again in 2019 to approximately the same level as 2017 and increased again in 2020. During each study year, the proportion of people who had heard about HPV exceeded the proportion that had heard about the HPV vaccine (Figure 1). As noted below, there were differences in awareness trends when examining respondents by sociodemographic characteristics.

### 3.1. Trends in HPV Awareness

In 2017, over 57% of all participants reported having knowledge of HPV. There were moderate increases in the percentage of respondents with awareness of HPV year over year, and by 2020, over 79% of respondents had heard about HPV overall (Table 1).

Between 2017 and 2020, non-Hispanic black participants, respondents with a high school level of education (or lower), those with an income less than $35,000, and participants between the ages of 50 and 64 experienced the largest increases in levels of awareness (97.2%, 77.7%, 44.5%, 40.4%, respectively).

**Figure 2:**
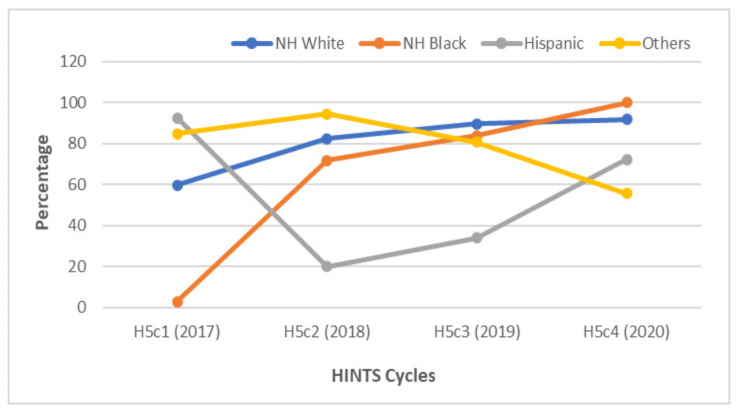
Awareness of HPV among gay and bisexual males according to race/ethnicity between 2017 and 2020. There was a large decrease in awareness about HPV among Hispanic GBM between 2017 and 2018. Annually, non-Hispanic (NH) white and black GBM gained awareness of HPV.

When examining respondents by race/ethnicity, HPV awareness consistently increased among non-Hispanic whites, from 59.7% in 2017 to 91.7% in 2020 (Figure 2). Awareness of HPV among Hispanic GBM was highest in 2017 at 92.4%. This was followed by a large drop to 19.9% in 2018 and a gradual increase to 72.2% by 2020.

**Figure 3:**
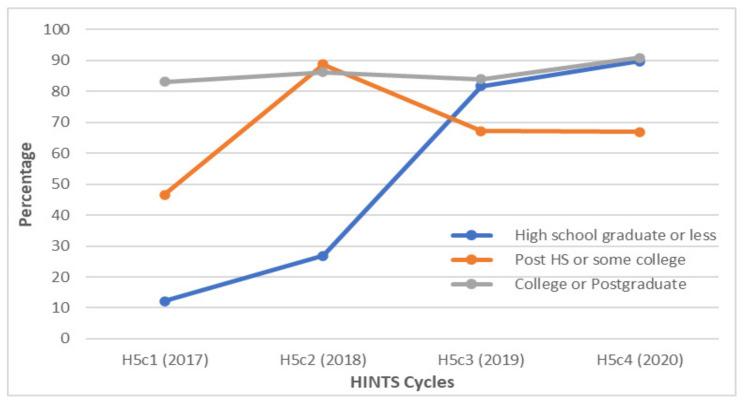
Awareness of HPV among gay and bisexual males according to education status between 2017 and 2020. Over 80% of GBM with college or postgraduate levels of education were aware of HPV each year. The largest increase in awareness occurred between 2018 and 2019 among GBM with a high school level of education or less; HS = high school.

When respondents were classified by education status, college graduates and postgraduates consistently had an awareness of HPV greater than 80% throughout the study period. However, the biggest change in the degree of HPV knowledge was among respondents with a high school education or less, who saw a 77.7% increase in awareness between 2017 and 2020 (Figure 3).

**Figure 4:**
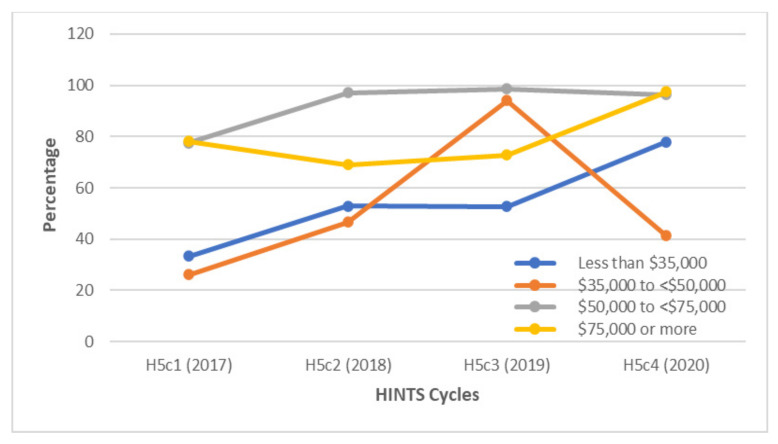
Awareness of HPV among gay and bisexual males according to income levels between 2017 and 2020. A higher percentage of GBM with incomes above $50,000 were aware of HPV compared to GBM with incomes below $50,000 in 2017, 2018, and 2020.

Differences in HPV awareness were also noted within socioeconomic strata. In 2017, approximately 78% of respondents who earned $50,000 or more had heard about the HPV vaccine, as opposed to only 33.4% and 26.2% of GBM who earned less than $35,000 and $35,000 to less than $50,000, respectively. By 2020, participants who earned between $50,000 to less than $75,000 and those who earned $75,000 or more had similar levels of awareness (Figure 4).

**Figure 5:**
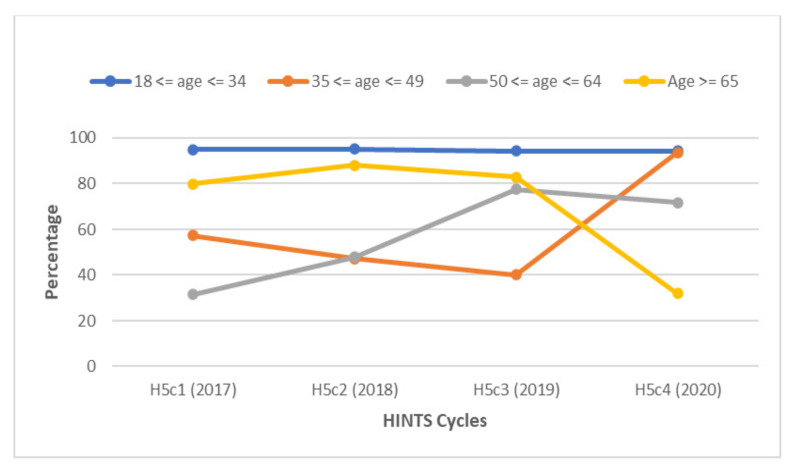
Awareness of HPV among gay and bisexual males according to age group between 2017 and 2020. The highest percentage of GBM with knowledge about HPV were between the ages of 18 and 34. Smaller proportions of GBM between the age of 35 and 64 were aware of HPV from 2017 to 2019.

When examining respondents by age group, participants aged 18–34 had the highest levels of awareness among the different age groups, with rates consistently above 90% throughout the study period (Figure 5). GBM between the ages of 35 and 64 had consistently lower rates of HPV awareness from 2017 to 2019. In 2020, over 50% more respondents between the ages of 35 to less than 50 were aware of HPV.

### 3.2. Trends in HPV Vaccine Awareness

In contrast to the HPV awareness levels, less than half of the total cohort were aware of the HPV vaccine in 2017. Overall, there was a 28.6% increase in HPV vaccine awareness between 2017 and 2020 (Table 2). 

Between 2017 and 2020, non-Hispanic black participants, respondents with a high school level of education (or lower), those with an income less than $35,000, and participants between the ages of 35 and 49 experienced the largest increases in awareness (97.2%, 56.7%, 48.2%, and 46.7%, respectively).

**Figure 6:**
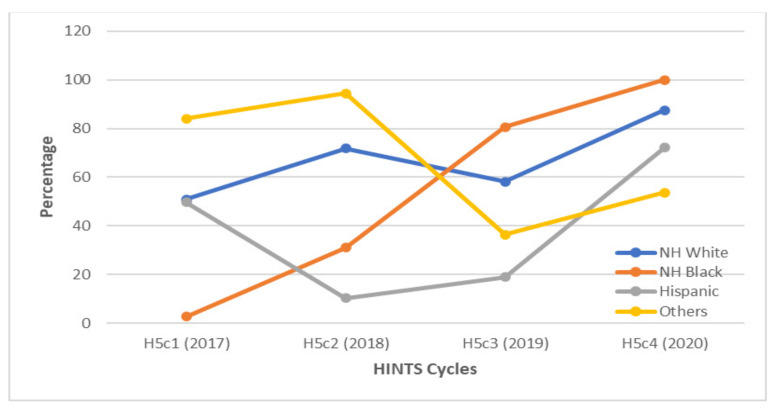
Awareness of the HPV vaccine among gay and bisexual males according to race/ethnicity between 2017 and 2020. There was a decrease in awareness about the HPV vaccine among Hispanic GBM between 2017 and 2018, followed by increased awareness during the rest of the study period. *NH = non-Hispanic*.

Similar to the trends of HPV awareness among GBM stratified by race, an initial decrease from 2017 to 2018 in HPV vaccine awareness was apparent among Hispanic GBM respondents, followed by a gradual increase to 72.2% by 2020 (Figure 6).

**Figure 7:**
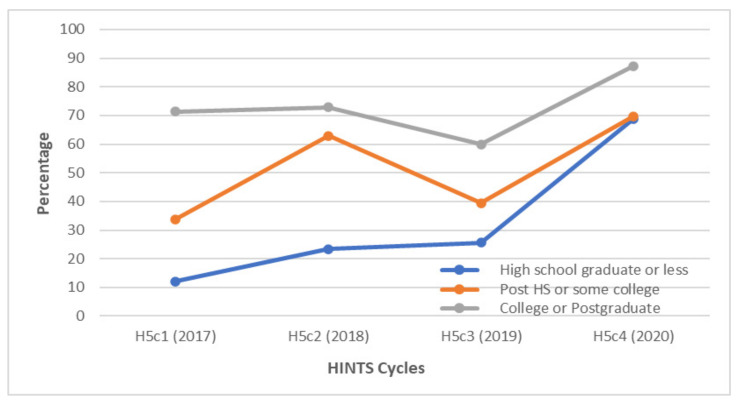
Awareness of HPV among gay and bisexual males according to education levels between 2017 and 2020. GBM with college or postgraduate levels of education were the most aware of the HPV vaccine each year. Levels of vaccine awareness fluctuated among GBM with post-high school or some college-level education; HS = high school.

When examining respondents by the level of education, GBM with high school education or less were the only group whose awareness of the HPV vaccine consistently increased over the study period (Figure 7). However, their levels of vaccine awareness remained lower than that of GBM with higher education levels. Those with post-high school or some college education had fluctuating levels of vaccine awareness over the study period.

**Figure 8:**
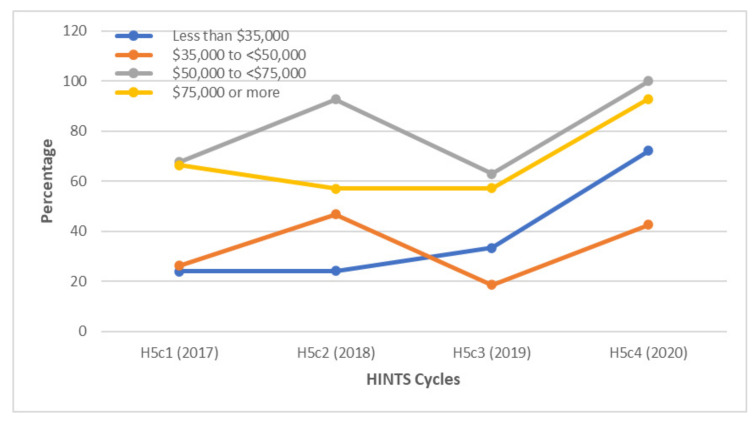
Awareness of the HPV vaccine among gay and bisexual males according to income levels between 2017 and 2020. A higher percentage of GBM with incomes above $50,000 were aware of the vaccine compared to GBM with incomes below $50,000 throughout the study period.

Additionally, GBM respondents with income levels above $50,000 had higher rates of awareness about the HPV vaccine than those with income levels below $50,000 from 2017 to 2020 (Figure 8).

**Figure 9:**
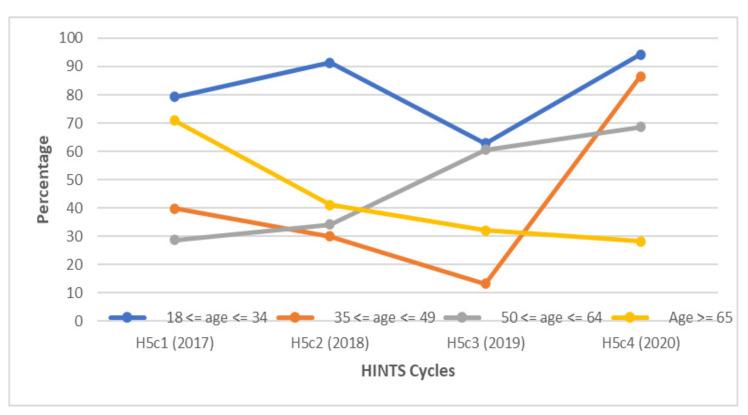
Awareness of the HPV vaccine among gay and bisexual males is illustrated according to specific age groups between 2017 and 2020. The highest percentage of GBM with knowledge about HPV over the entire study period were between the ages of 18 and 34. A gradual decline in HPV vaccine awareness was observed in GBM aged 65 and older.

In 2017, 70.8% of GBM aged 65 years or older were aware of the HPV vaccine. However, this sharply dropped to 41% in 2018 and continued to decline to 28.2% by 2020 (Figure 9). Respondents aged 18 to 34 had higher levels of awareness each year compared to older GBM, including in 2019, when there was a 28.4% decline in their level of awareness from the prior year.

## 4. Discussion

This study demonstrates that awareness of HPV and the HPV vaccine among gay and bisexual males (GBM) in the US increased overall between 2017 and 2020. However, the results varied differently when examining the data by sociodemographic characteristics.

Healthcare providers (HCP) play a very important role in HPV awareness and vaccination. HCP recommendation is generally the most influential factor affecting HPV vaccination in males [6,22,23]. Similarly, HCP recommendation has also been identified in other studies as the strongest predictor of HPV vaccination uptake in men who have sex with men and transgender women [13,24]. In one study, MSM who had received a provider recommendation were 40 times more likely to have been vaccinated [24]. In another study, 83% of MSM received an HPV vaccination after receiving a provider recommendation, while only 5% of MSM who did not receive a provider recommendation pursued vaccination [6]. In our study, non-Hispanic white and black GBM demonstrated a significant increase in awareness of both HPV and the HPV vaccine by 2020. Hispanic GBM, on the other hand, had lower levels of awareness of HPV than these two groups from 2018 to 2020. One of the multifactorial mechanisms underlying this disparity could be the racial differences in the level of trust in information from healthcare providers [22]. Hispanics have been found to be the least likely racial or ethnic group to see a medical provider, and Hispanic men are the least trusting of information about cancer from HCPs [22,25]. Hispanic MSM were also found to be less likely to be vaccinated against HPV than white or black GBM in another study [24]. However, improving relationships and perceptions of providers is not the only factor that needs to be addressed. There is also a need to improve the rates of HCP recommendation of the HPV vaccine and decrease missed opportunities for HPV vaccination at medical visits for vaccine-eligible GBM [6,26]. A recent study found that the prevalence of HCP recommendation of the HPV vaccine was roughly twice as high in women than in men [18]. Another study found primary care physicians viewed HPV vaccine discussions as burdensome and recommended the HPV vaccine less strongly than other adolescent vaccines [27]. In one study, 40% of HPV vaccine-eligible GBM had visited their HCP within the previous year, but only 11% had received an HPV vaccine recommendation from their provider [6]. There is, therefore, a need for the employment of strategies to support physicians in recommending the HPV vaccine with more efficiency and confidence [27].

In our study, college graduates and postgraduates had consistently higher levels of HPV vaccine awareness throughout the study period compared to respondents with lower levels of education. This is in keeping with the results of other studies with similar findings [14,22]. Other studies also identified GBM with a college degree as having higher HPV vaccination rates than respondents with lower education levels [6,13]. Most universities provide easily accessible on-site medical facilities for students, offering HPV vaccination and education, among other preventive services [28]. However, our study cannot infer whether the high levels of HPV and HPV vaccine awareness in GBM with a college degree correlate with higher HPV vaccination rates or intent in this subgroup. A recent study showed that while 77% of college men had heard about HPV, only 28% of this group had received at least one dose of the HPV vaccine [28]. In another study, 79.5% of college men had heard about HPV and the HPV vaccine, but only 28.4% of them were more likely to intend to get vaccinated [29]. Therefore, the burden of improving HPV awareness and vaccination rates should not rest exclusively on HCPs. Given their easy access to these students, campus facilities and student organizations are uniquely positioned to raise HPV awareness and vaccination rates among college students [29]. A study identified the internet and school as the most common sources of information about HPV and the HPV vaccine in male college students [30]. Another study reported an association between intent and social support, finding that males were more likely to intend to get vaccinated if they felt greater social support [29,31]. Therefore, utilizing these resources may help bridge the gap between awareness and vaccination intent and uptake [29].

Under-education and low socioeconomic status often co-exist and are associated with lower awareness of the HPV vaccine and lower vaccination rates [14]. It is important to employ a multi-pronged approach while addressing deficits in HPV and HPV vaccine awareness [14]. In our study, respondents with annual household incomes of at least $50,000 had higher HPV and HPV vaccine awareness each year compared to GBM with lower income levels. Findings in other studies could perhaps explain our findings. GBM with minimum household incomes of $50,000 have been found to disclose their sexual orientation to their HCP at higher rates than GBM who earn lower incomes [6,26]. While HPV-related discussions should ideally be had, regardless of sexual orientation, disclosure of sexual orientation by GBM to HCPs may prompt more in-depth discussions related to HPV. In one study, MSM who had disclosed their sexual orientation were at least twice as likely to receive pertinent preventive healthcare measures than those who did not [32]. Therefore, campaigns encouraging GBM to disclose their sexual orientation and emphasizing why such disclosure is salient should be promoted [32]. While community organizations serving sexual minorities often have referral information for HCPs known to be sensitive to their unique needs, widely disseminating this information would benefit other individuals who have little to no contact with these organizations [32]. HCPs also need to create an environment where GBM are comfortable disclosing their sexual orientation to limit missed opportunities for HPV vaccine recommendation and administration [6,26]. Beyond the issue of disclosing sexual orientation, there is also a need to target pertinent low-income groups with tailored educational resources to complement larger-scale state/national level efforts [14]. For example, a study targeting a low-income Hispanic medically underserved community in Southern California succeeded in remarkably improving HPV vaccination awareness, attitudes, and intention through the use of a fotonovella [14,33]. Utilizing similar tailored interventions using characteristics unique to the relevant GBM sub-population may help address barriers beyond the reach of larger-scale strategies [14].

Participants aged 18–34 years had the highest levels of HPV and HPV vaccine awareness each year. On the other hand, respondents aged 65 years or older had declining HPV vaccine awareness levels annually and, by 2020, had the lowest level of all the age groups. These findings are consistent with other studies [14,34]. One suggested explanation is that younger people may have been targets of awareness campaigns in the years following the introduction of the HPV vaccine [14]. However, the lower levels of HPV vaccine awareness among older males may be problematic, as these individuals could be a valuable source of information for younger males.

To our knowledge, this is the first study focused on assessing the trends in HPV and HPV vaccine awareness in GBM in the US over time. Our study had some limitations. First, our study was limited to male respondents who self-identified as gay or bisexual. This may be problematic as not all MSM self-identify as gay or bisexual [6]. Additionally, our small sample size and the cross-sectional nature of the HINTS study may limit the generalizability of our findings. Additionally, the self-reported data used in our study could be influenced by social desirability bias, which would negatively affect the accuracy of the analysis [22,29].

It is encouraging that awareness of HPV and the HPV vaccine increased overall between 2017 and 2020. Further studies will be needed to evaluate whether the HPV vaccination rates among GBM have also increased over time as well. However, the fluctuations in levels of awareness, and even decline in some sociodemographic groups in our study, highlight the need for more interventions aimed at improving HPV-related awareness among GBM in the US.

## Figures and Tables

**Table 1:** Awareness of HPV among gay and bisexual males in the US.

	2017 *N* = 61			2018 *N* = 60			2019 *N* = 100			2020 *N* = 67		
	%	(95% CI)		%	(95% CI)		%	(95% CI)		%	(95% CI)	
**Overall**	57.6	53.3	61.8	67.6	61.9	73.4	76.8	74.8	78.9	79.7	77.2	82.1
**Race/Ethnicity**												
NH White	59.7	55.3	64.1	82.4	77.4	87.5	89.6	88.6	90.7	91.7	89.9	93.5
NH Black	2.73	0	41.9	71.8	64.1	79.5	83.9	80.8	87.1	100	100	100
Hispanic	92.4	88.7	96.2	19.9	1.69	38.1	34.0	26.0	41.9	72.2	61.6	82.8
Others	84.7	76.7	92.6	94.5	78.3	100	80.7	79.1	82.4	55.5	41.9	69.0
**Education**												
H. school grad or less	12.1	1.96	22.3	26.8	17.2	36.4	81.7	79.5	83.8	89.8	81.8	97.8
Post HS or some college	46.6	39.3	54.0	88.8	84.3	93.2	67.2	61.9	72.6	66.9	61.5	72.3
College or postgraduate	83.1	80.2	86.0	86.2	84.1	88.3	84.0	83.0	85.1	90.9	89.0	92.8
**Household Income**												
Less than $35,000	33.4	24.4	42.5	52.9	46.9	58.9	52.7	49.8	55.6	77.9	72.8	83.1
$35,000 to <$50,000	26.2	13.0	39.4	46.7	27.3	66.1	94.1	93.3	94.9	41.4	34.4	48.4
$50,000 to <$75,000	77.5	68.3	86.6	97.2	95.1	99.3	98.6	98.4	98.9	96.3	93.7	99.0
$75,000 or more	78.1	73.0	83.3	69.1	59.6	78.7	72.9	68.9	76.8	97.4	96.2	98.5
**Age**												
18 ≤ age ≤ 34	95.0	92.8	97.3	95.1	92.5	97.8	94.2	93.9	94.5	94.2	92.4	95.9
35 ≤ age ≤ 49	57.3	49.9	64.6	47.2	27.8	66.6	40.1	30.1	50.1	93.7	90.6	96.9
50 ≤ age ≤ 64	31.4	25.1	37.7	47.9	40.2	55.6	77.4	75.5	79.3	71.8	65.1	78.5
Age ≥ 65	79.9	75.2	84.7	88.0	84.1	91.9	82.9	79.9	85.9	32.2	26.3	38.2

**Table 2:** Awareness of the HPV vaccine among gay and bisexual males in the US.

	2017			2018			2019			2020		
	%	(95% CI)		%	(95% CI)		%	(95% CI)		%	(95% CI)	
**Overall**	48.0	44.4	51.6	53.0	46.9	59.2	47.9	46.5	49.3	76.6	73.8	79.5
**Race/Ethnicity**												
NH White	50.9	46.5	55.2	71.8	65.1	78.4	58.1	56.9	59.3	87.6	85.2	89.9
NH Black	2.73	0	41.9	31.1	20.2	42.0	80.6	78.8	82.4	100	100	100
Hispanic	49.7	37.9	61.4	10.2	0.818	19.6	18.9	14.5	23.3	72.2	61.6	82.8
Others	84.1	76.0	92.2	94.5	78.3	100	36.4	32.7	40.2	53.6	40.0	67.2
**Education**												
High school graduate or less	12.1	1.96	22.3	23.4	11.2	35.6	25.6	23.3	27.9	68.8	45.2	92.3
Post HS or some college	33.8	27.4	40.2	62.9	50.3	75.5	39.4	35.9	43.0	69.8	64.6	74.9
College or Postgraduate	71.4	67.0	75.7	72.9	68.4	77.5	59.9	58.6	61.2	87.3	85.1	89.5
**Household Income**												
Less than $35,000	24.0	17.0	31.1	24.1	19.6	28.7	33.3	31.3	35.2	72.2	66.1	78.3
$35,000 to <$50,000	26.2	13.0	39.4	46.7	27.3	66.1	18.6	15.7	21.4	42.6	35.5	49.7
$50,000 to <$75,000	67.6	57.3	77.8	92.8	87.6	97.9	63.0	60.8	65.2	100	100	100
$75,000 or more	66.5	61.0	72.0	57.0	47.9	66.2	57.2	53.9	60.4	92.9	91.1	94.6
**Age**												
18 ≤ age ≤ 34	79.2	73.8	84.7	91.3	87.3	95.2	62.9	61.0	64.7	94.2	92.4	95.9
35 ≤ age ≤ 49	39.8	33.3	46.3	30.0	16.6	43.4	13.1	9.64	16.5	86.5	81.0	92.1
50 ≤ age ≤ 64	28.6	22.7	34.4	34.2	28.7	39.8	60.6	58.5	62.6	68.7	61.8	75.5
Age ≥ 65	70.8	63.4	78.2	41.0	33.7	48.2	32.1	29.0	35.1	28.2	22.8	33.6

## Data Availability

The data used in this study are available, upon request, from the United States National Cancer Institute.
